# Intraoperative nasogastric tube in robotic thoracic surgery: lower gastrointestinal risk without added respiratory risk

**DOI:** 10.1007/s11701-025-02458-3

**Published:** 2025-06-23

**Authors:** Sara Volpi, Akshay Patel, Giulia Fabbri, Alex Smith, Federico Femia, George Christodoulides, Craig Johnstone, Tom Routledge, Andrea Bille

**Affiliations:** 1 Department of Thoracic Surgery, Guy’s Hospital, Great Maze Pond, London, SE1 9RT UK; 2 Institute of Immunology and Immunotherapy, College of Medical and Dental Sciences, University of Birmingham, Edgbaston, B152TT England, UK; 3 Department of Thoracic Anaesthesia, Guy’s Hospital, London, UK

**Keywords:** Robotic thoracic surgery, Intra-abdominal complications, Gastrointestinal complications, Ileus, Bowel obstruction, Respiratory complications, Anatomical lung resection

## Abstract

Gastrointestinal (GI) complications are a leading cause of postoperative morbidity in thoracic surgery patients. Robotic thoracic surgery, with CO2 insufflation, may heighten GI risks, such as ileus or bowel obstruction. Intraoperative nasogastric tube (iNGT) use has the potential to mitigate these risks by reducing gastric content and preventing aspiration. This retrospective study evaluated the impact of iNGT on postoperative GI and respiratory complications in 718 patients undergoing robotic anatomical lung resections from 2017 to 2022. Patients were divided into iNGT (*n* = 450) and non-iNGT (*n* = 268) groups. GI complications were significantly lower in the iNGT group (1.8 vs. 10%, *p* < 0.0001), with an adjusted odds ratio of 5.85 for non-iNGT patients. No significant difference was observed in respiratory complications (19.3 vs. 18.6%, *p* = 0.82). These findings suggest that iNGT reduces GI complications without increasing respiratory risks, supporting its selective use in robotic thoracic surgery.

## Introduction

Gastrointestinal (GI) complications are the second leading cause of mortality after respiratory complications in thoracic surgery patients [1]. Reduced GI motility, which can result in ileus, bowel obstruction, or pseudo-obstruction, may lead to aspiration and subsequent respiratory issues. Robotic thoracic surgery, involving CO_2_ insufflation, might increase the risk of these complications [2].

Intraoperative nasogastric tube (iNGT) use during robotic thoracic surgery can mitigate both GI and respiratory complications by reducing gastric content, preventing regurgitation and aspiration, and improving surgical access through decompression of the stomach. This study aims to evaluate the impact of iNGT on the incidence of postoperative GI and respiratory complications in patients undergoing robotically assisted anatomical lung resection.

## Methods

We retrospectively analyzed patients undergoing elective robotic anatomical lung resection at our center between 2017 and 2022. iNGT insertion (14–16 French) was based on the anaesthetist’s practice, not patient-specific criteria hence no systematic bias in selecting higher risk patients for iNGT. All patients followed standard fasting protocols preoperatively (i.e., nil by mouth for at least 6 h for solids and 2 h for clear fluids prior to induction), with no use of mechanical bowel preparation. Patients undergoing segmentectomy or lobectomy were included, while those who required conversion to thoracotomy or had previous abdominal or head and neck surgery were excluded. The iNGT was removed immediately after surgery upon extubation in the operating theater in all cases to allow proper postoperative mobilization and engagement in physiotherapy. With regards to the diagnosis of complications, this was based on clinical criteria primarily with radiological confirmation or support. Clinical signs, such as abdominal distension, vomiting, and failure to tolerate diet, prompted abdominal imaging (abdominal X-ray or CT). Routine chest X-rays were performed to assess postoperative atelectasis, consolidation, and the presence of pleural effusion.

### Statistical analysis

Continuous data were compared using t tests or Wilcoxon rank-sum tests, while categorical data were analyzed using Chi-square or Fisher’s exact tests. Multivariable logistic regression assessed the impact of iNGT on outcomes, incorporating age, gender, BMI, transfer factor, and laterality of surgery. We assessed for multicollinearity using Variance Inflation Factors (VIF), and no significant collinearity was observed (all VIFs < 2).

## Results

Between 2017 and 2022, 718 patients met the inclusion criteria, with 450 (63%) receiving iNGT (group A) and 268 (37%) not receiving it (group B) (Table 1). Gastrointestinal (GI) complications, including ileus, bowel obstruction, and pseudo-obstruction, were significantly lower in group A (1.8%) compared to group B (10%, *p* < 0.0001). After adjustment, the odds ratio for GI complications was 5.85 times higher in group B (*p* < 0.001). MV analysis identified iNGT use as the sole significant independent predictor of postoperative GI complications. However, there was no significant difference in the incidence of respiratory complications between groups (19.3% in group A vs 18.6% in group B, *p* = 0.82). The primary GI complication was ileus, with no patient requiring abdominal surgery. The difference in GI complications did not translate into a statistically significant difference between groups in terms of postoperative length of stay (LOS), ICU admission rates, or readmission to hospital.

**Table 1:** Basic demographic and early postoperative outcome data

	Patients with iNGT (*n* = 450) group A	Patients without iNGT (*n* = 268) group B	*p* Value
Age (years)	69 (SD 10)	70 (SD 10)	0.04
Gender	Male 186 (41%)	Male 102 (38%)	0.35
	Female 264 (59%)	Female 166 (62%)	
Preop FEV1 (%)	92% (IQR 77–109)	89% (IQR 74–104)	0.30
Preop TLCO (%)	76% (IQR 63–88)	76% (IQR 63–88)	0.54
Laterality	Right: 264	Right: 162	0.64
	Left: 186	Left: 106	
BMI	27 (SD 6)	28 (SD 6)	0.031
GI complications	8 (1.8%)	27 (10%)	< 0.0001
Respiratory complications	87 (19.3%)	50 (18.6%)	0.82
30-day mortality	7 (1.6%)	1 0.4%)	0.14

## Discussion

This study demonstrates that iNGT use during robotic lung resections significantly reduces postoperative GI complications, particularly ileus, bowel obstruction, and pseudo-obstruction. No significant effect on respiratory complications was observed. Multivariable analysis did not show any significant relationship between iNGT use and postoperative respiratory outcomes, and factors like age, gender, preoperative lung function, BMI, and laterality of surgery did not influence the incidence of complications.

While a few studies have focused on iNGT use in robotic thoracic surgery, iNGT has been established in other surgical contexts to reduce gastric distension and prevent nausea, vomiting, and regurgitation. Gastric overdistension which can be induced by CO₂ insufflation results in a decrease in intragastric pressure that may compromise bowel motility. By decompressing the stomach, iNGT helps preserve diaphragmatic movement and gastrointestinal peristalsis, which may reduce the incidence of ileus and pseudo-obstruction [3]. Furthermore, CO_2_ insufflation is linked to complications such as respiratory acidosis and CO_2_ embolism which can be life-threatening and must be considered alongside other critical diagnoses, such as tension pneumothorax and cardiac tamponade [4, 5].

However, some studies have raised concerns about the increased risk of respiratory complications, such as pneumonia, due to the loss of esophageal sphincter integrity with prolonged NGT use [5]. Contrary to concerns about respiratory risks, our findings support iNGT’s use during surgery only, with immediate removal postoperatively. By reducing intraoperative GI complications without increasing respiratory risks, iNGT may offer significant benefits for patients undergoing robotic lung resections [6, 7]. However, iNGT use should be individualized based on patient factors, such as pre-existing GI conditions or surgery complexity.

## Conclusion

Our study found that iNGT significantly reduces postoperative GI complications without increasing respiratory complications in patients undergoing robotically assisted anatomical lung resection. These findings support routine iNGT use during such procedures, particularly in patients at higher risk for postoperative GI issues. Future research is needed to validate these results in a larger patient cohort and to explore the potential benefits of iNGT in other types of thoracic surgeries.

## Data Availability

No datasets were generated or analysed during the current study.
